# The Physicians Visiting List (Lindsay & Blakiston)

**Published:** 1885-11

**Authors:** 


THE PHYSICIANS VISITING LIST—(Lindsay & Blakiston) for 1886. Thirty-fifth year of its publication. Publishers: P. Blakiston, Son & Co., Philadelphia.

Annually this useful Visiting List makes its appearance, every edition showing an improvement on the former one. The present edition contains a Calendar, List of Poisons and Antidotes, Dose Tables rewritten in accordance with the sixth revision of the U. S. Pharmacopœia, Marshall Hall's Ready Method in Asphyxia, Lists of New Remedies, Sylvester's Method for producing Artificial Respiration, with Illustrations; Diagram for Diagnosing Diseases of Heart, Lungs, etc., etc., different sizes and also a perpetual edition as to dates are published of this list, and every copy contains a superior pencil with a nickel tip.

